# Utilization and Safety of Concurrent Use of Abemaciclib and Radiation Therapy Among Patients With HR+, HER2− Metastatic Breast Cancer in the Real-World Setting

**DOI:** 10.1016/j.adro.2025.101992

**Published:** 2025-12-31

**Authors:** Wambui Gathirua-Mwangi, Sangmi Kim, Holly Martin, Tasneem Lokhandwala, Shen Zheng, Eileen Farrelly, Erich Brechtelsbauer, Sarah Rybowski, Kamran A. Ahmed

**Affiliations:** a Eli Lilly and Company, Indianapolis, Indiana; b Cencora, 1 West First Avenue, Conshohocken, Pennsylvania; c TechData Service Company LLC, King of Prussia, Pennsylvania; d H. Lee Moffitt Cancer Center & Research Institute, Tampa, Florida

## Abstract

**Purpose:**

Real-world data on tolerability of concurrent radiation therapy (RT) and abemaciclib are limited. This study described real-world utilization and safety of concurrent RT and abemaciclib in patients with hormone receptor-positive (HR+) and human epidermal growth factor receptor 2-negative (HER2−) metastatic breast cancer (MBC).

**Methods and Materials:**

This retrospective study accessed data from the Flatiron Health United States nationwide deidentified electronic health records-derived longitudinal database. Abemaciclib initiation date was the index date (September 2017-September 2021). Concurrent RT was defined as receipt of any RT with ≥1 day of overlap with abemaciclib therapy. The minimum follow-up time was 90 days. Patient characteristics, treatment patterns, and real-world adverse events (rwAEs) were presented descriptively; Kaplan–Meier methods were used to assess time to treatment discontinuation.

**Results:**

This study included 174 female patients with a median follow-up time of 17.5 (IQR, 10.1-26.5) months. The median age was 63.0 (IQR, 54.0-71.0) years, 8.0% had Eastern Cooperative Oncology Group Performance Status ≥ 2 at index and 31.0% had MBC. Prior to abemaciclib use, 20.1% and 28.2% of patients had chemotherapy or other CDK4/6 inhibitors, respectively. About half of the patients (47.7%) received abemaciclib in combination with fulvestrant, and 76.4% initiated abemaciclib at 150 mg twice daily. Overall, 151 patients (86.8%) initiated RT at or after initiation of abemaciclib. During concurrent RT and abemaciclib use, 76.4% of patients had no dose change in abemaciclib; 16.7% and 2.3% had a dose hold or dose reduction, respectively; 4.0% discontinued abemaciclib. The incidence of rwAEs during concurrent abemaciclib + RT were diarrhea (73.0%), fatigue (62.6%), rash (27.6%), and neutropenia (23.0%). The median (95% CI) time to treatment discontinuation was 368 (290-516) days.

**Conclusions:**

Most patients did not require a dose modification or interruption with concurrent RT and abemaciclib. These findings suggest that the addition of RT to abemaciclib therapy is well tolerated in patients with HR+, HER2− MBC.

## Introduction

Breast cancer is the most common cancer diagnosed in women and the leading cause of cancer-related deaths in the United States (US).^1^ A majority of breast cancers (BCs) are of the molecular subtype hormone receptor-positive (HR+), human epidermal growth factor receptor 2 negative (HER2−), which is associated with a more favorable prognosis and survival rates versus other subtypes.^2^^,^^3^ Cyclin-dependent kinase 4/6 inhibitors (CDK4/6i) are the current standard of care for the treatment of HR+, HER2− advanced or metastatic BC (MBC).^4^ Between 2015 and 2017, the US Food and Drug Administration approved 3 CDK4/6i – palbociclib, ribociclib, and abemaciclib for use in the metastatic setting.^5^, ^6^, ^7^ To date, abemaciclib is the only CDK4/6i approved for the adjuvant treatment of HR+, HER2− in node-positive, early BC at high risk of recurrence, and as monotherapy for patients with HR+, HER2- MBC with disease progression following endocrine therapy (ET) and prior chemotherapy in the metastatic setting.^8^, ^9^, ^10^, ^11^

Radiation therapy (RT) is a widely used palliative treatment option in MBC.^12^^,^^13^ For patients with limited or resected brain metastases, stereotactic radiosurgery (SRS) is recommended. Whole-brain RT (WBRT) is recommended as a palliative procedure when brain metastases that are not amenable to SRS are present.^14^ In addition, stereotactic body radiation therapy (SBRT) has an evolving role in the management of oligometastatic BC.^15^ However, RT administration can be associated with adverse events (AEs) such as suppression of the bone marrow,^16^ and in some patients with MBC, gastrointestinal AEs have also been reported.^17^

Preclinical studies have shown that CDK4/6i sensitized HR+, HER2− BC cell lines to RT and could potentially augment the therapeutic effect of RT.^18^ Limited data from retrospective studies showed that over a tenth of patients who received concurrent RT and CDK4/6i (mostly palbociclib) had severe toxicities,^19^ although some data from Europe have suggested that concurrent RT and CDK4/6i use was not associated with higher AEs (grade ≥ 3).^20^ Case reports of patients who were administered concurrent RT and palbociclib have also reported higher toxicity with this combination.^17^ A few retrospective studies assessed the safety of concurrent RT and CDK4/6i, albeit with a small sample size of patients, and reported colitis (16.7%),^21^ radionecrosis (5%),^22^ and grade 3 hematological toxicities (10.5%).^23^ Compared to palbociclib or ribociclib, abemaciclib has a greater affinity for CDK4 versus CDK6 resulting in different safety profiles.^24^ Nonhematological toxicities such as diarrhea and fatigue are more commonly seen with abemaciclib use and hematological toxicities (neutropenia) are lower versus palbociclib or ribociclib.^25^ Given that abemaciclib use has been widely adopted since its Food and Drug Administration approval in 2017, and its expanding indication since then, an evaluation of the safety of its concurrent use with RT in the MBC population is warranted. No clinical trials or real-world studies so far have elucidated the safety of administering RT concurrently with abemaciclib in patients with MBC. Therefore, this study aimed to describe patient characteristics, treatment patterns, and safety of concurrent abemaciclib and RT using data from real-world patients with HR+, HER2− MBC.

## Methods and Materials

### Study design

This is a retrospective cohort study of US patients diagnosed with HR+, HER2- MBC who received RT concurrently with abemaciclib. Data were accessed from the Flatiron Health database and the data originated from approximately 265 to 280 US cancer clinics (∼800 sites of care). The study period was from September 1, 2017 to December 31, 2021 (Fig. E1).

### Data source

The Flatiron Health database is a nationally representative, electronic health records (EHR)-derived longitudinal, deidentified database. The majority of patients in the database originated from community oncology settings.^26^^,^^27^ Flatiron Health has access to both patient-level structured and unstructured data contained within the EHR, curated via technology-enabled abstraction, which are processed and merged into a single database. The database includes EHR data elements such as patient demographics, clinical characteristics, treatments received, treatment outcomes, and clinician-documented treatment response.

This study was conducted in accordance with the ethical principles that have their origin in the Declaration of Helsinki, and are consistent with Good Pharmacoepidemiology Practices, and applicable laws, and regulations in the US. Provisions were in place to prevent reidentification of patient-level data to protect patients’ confidentiality. The patient data used in this noninterventional study were deidentified in compliance with Health Insurance Portability and Accountability Act regulations. This observational study only used previously collected data, did not impose any form of intervention, and deidentified the data to protect participant privacy. Therefore, a formal Consent to Release Information form was not required.

### Patient population

The study population included adult patients (≥18 years old) with an International Classification of Diseases, Ninth or Tenth Revision, Clinical Modification (ICD-9-CM or ICD-10-CM) diagnosis of BC (ICD-9-CM 174.x or 175.x or ICD-10-CM C50x), having at least 2 documented clinical visits on different days in the Flatiron Health database, evidence of stage IV de novo or recurrent MBC with a metastasis diagnosis date on or after January 1, 2011, and inclusion in the probabilistic sample of patients queued for review through abstraction implemented by Flatiron Health. In addition, patients having an abemaciclib initiation date (index date) between September 1, 2017 and September 30, 2021 (index period; Fig. E1), a diagnosis of HR+, HER2− any time within 30 days following index date, and evidence of concurrent RT for MBC were included. Concurrent RT was defined as receipt of any RT with at least 1 day of overlap with the abemaciclib treatment span. Patients were excluded from this study if there was evidence of a primary malignancy other than BC, subjects treated with a clinical study drug or HER2+ directed therapies in combination with abemaciclib in the metastatic setting, evidence of abemaciclib treatment for >14 days prior to MBC diagnosis date, or with <7 days of abemaciclib treatment; or patients with a follow-up of <90 days except due to death (Fig. E2).

### Outcomes and definitions

In this study, we described the incidence of real-world adverse events (rwAEs) among abemaciclib treated patients with HR+, HER2− MBC who received concurrent RT. A rwAE was defined as an unfavorable sign, symptom, or diagnosis that either begins or worsens (if the diagnosis was present at baseline) during abemaciclib treatment with overlapping RT spans. The rwAEs of interest were prespecified based on clinical relevance and input from key thought leaders. These rwAEs were neutropenia, diarrhea, stomatitis, rash, fatigue, pneumonitis, and colitis. Trained medical data abstractors abstracted the prespecified rwAEs from unstructured data using rules delineated by Flatiron Health. Clinicians verified and validated the abstracted data. The first documented occurrence of these prespecified rwAEs from index date up to and including 30 days post-abemaciclib discontinuation were extracted from patient records.

Time to abemaciclib discontinuation (TTD) was defined as the time from the index date to abemaciclib discontinuation date or death. Patients who remained on abemaciclib treatment at the end of follow-up were censored at the last visit date or study end date, whichever was earlier. Reasons for abemaciclib discontinuation were described. Other variables assessed included characterizing concurrent abemaciclib and RT users and associated treatment patterns (abemaciclib regimens, duration of abemaciclib treatment, starting dose, time to dose change, type of RT, RT dose, dose fraction, and RT site).

### Statistical analysis

Descriptive analyses were performed to assess the incidence of prespecified rwAEs, as well as to describe demographic and clinical variables, treatment patterns, and proportion of patients with a change in abemaciclib dose. Data were reported as frequencies and percentages for categorical variables and means with SDs, medians, and IQRs were reported for continuous variables. TTD was assessed using Kaplan–Meier methodology and medians and 95% CIs were reported.

## Results

### Patient characteristics

The study cohort comprised 174 patients with HR+, HER2− MBC who received concurrent abemaciclib and RT. The median follow-up time from abemaciclib initiation was 17.5 months (IQR, 10.1-26.5 months). The median age was 63 years (IQR, 54.0-71.0 years). A majority of the patients were White (58.6%) and Black/African Americans comprised 12.1%; 92.5% accessed cancer care at community practices. Approximately 31.0% of the patients were diagnosed with de novo MBC, and 83.3% were classified as postmenopausal. A majority of patients (81.6%) had Eastern Cooperative Oncology Group performance status 0 to 1 and 8.0% had Eastern Cooperative Oncology Group performance status ≥ 2 at index (Table 1). The median time from MBC diagnosis to abemaciclib initiation was 2.1 months (IQR, 0.8-13.6 months) and 66.7% of patients had 1 to 2 metastatic sites at index date; metastasis to the bone was the most common site (90.8%), followed by visceral (43.7%), and lung (24.1%) (Table 1).

**Table 1:** Baseline characteristics of patients with HR+, HER2− MBC who used concurrent abemaciclib and RT

Variables	Overall N = 174
Follow-up time (mo), median (IQR)	17.5 (10.1, 26.5)
Demographics	
Age (y), median (IQR)	63.0 (54.0, 71.0)
Race, n (%)	
White	102 (58.6)
Black	21 (12.1)
Asian	6 (3.4)
Other*	28 (16.1)
Missing/unknown	17 (9.8)
Practice type, n (%)	
Academic	6 (3.5)
Community	161 (92.5)
Unknown	7 (4.0)
Clinical characteristics	
Menopausal status, n (%)†	
Pre/peri-menopausal	18 (10.3)
Postmenopausal	145 (83.3)
Unknown‡	11 (6.3)
Stage at initial diagnosis, n (%)	
Stage I	18 (10.3)
Stage II	55 (31.6)
Stage III	37 (21.3)
Stage IV	54 (31.0)
Not documented	10 (5.7)
ECOG performance status, n (%)	
0-1	142 (81.6)
2-4	14 (8.0)
Unknown	18 (10.3)
Charlson Comorbidity Index, n (%)	
0	116 (66.7)
1	29 (16.7)
2	16 (9.2)
≥3	13 (7.5)
Number of metastatic sites at index date, n (%)	
1-2	116 (66.7)
≥3	58 (33.3)
Metastasis sites at index date, n (%)	
Bone	158 (90.8)
Visceral	76 (43.7)
Lung	42 (24.1)
Liver	39 (22.4)
Brain	33 (19.0)
Prior therapy in metastatic setting, n (%)	
Chemotherapy	35 (20.1)
Hormone therapy	83 (47.7)
Targeted and immunotherapy	51 (29.3)
CDK4/6i	49 (28.2)
Other§	11 (6.3)
Time from MBC diagnosis to abemaciclib initiation (mo), median (IQR)	2.1 (0.8, 13.6)

*Abbreviations:* CDK4/6i = cyclin-dependent kinase 4/6 inhibitors; ECOG = Eastern Cooperative Oncology Group; HR+, HER2− = hormone receptor-positive, human epidermal growth factor receptor 2 negative; MBC = metastatic breast cancer; N = total number of patients; n = number of patients for the variable; RT = radiation therapy.

⁎ Includes American Indian or Alaska Native, Hawaiian or Pacific Islander, and multiple categories.

† Females >60 years at index were assigned as postmenopausal and if <60 years, if they underwent bilateral oophorectomy before or up to 30 days of using any abemaciclib-containing therapy.

‡ Menopausal status not documented.

§ Includes phosphoinositide 3-kinase inhibitors, the mammalian target of rapamycin inhibitors, and clinical trial participants.

### Abemaciclib utilization and concurrent RT treatment patterns

Prior to initiating abemaciclib, 47.7% of patients had received ET, 20.1% had received chemotherapy, and 28.2% had received palbociclib or ribociclib in the metastatic setting (Table 1). Most patients received abemaciclib in either the first (47.1%) or second (31.0%) lines of therapy. Overall, 21 (12.1%) patients used abemaciclib as monotherapy, and 142 (81.6%) used abemaciclib in combination with ET. Demographic and clinical characteristics of the patients by the abemaciclib treatment regimens are presented in Table E1.

Abemaciclib was initially administered at the standard 150 mg twice daily dose in 133 (76.4%) patients and 17 (9.8%) initiated abemaciclib at a dose of 100 mg twice daily. Overall, 79 (45.4%) patients had a dose modification, of these 61 patients (77.2%) had a dose reduction of ≥50 mg daily and 7 (8.9%) had a dose increase of ≥50 mg daily. The median time to dose modification was 65.0 days (IQR, 37.0-173.0 days).

The most common sites of RT were spine (31.0%); bone, other (19.0%), brain (17.2%), and hip and pelvis (both 13.8%) (Table 2). Table E2 summarizes the sites per abemaciclib treatment regimens. 3D-Conformal radiotherapy (13.8%) and SBRT (11.5%) were the most common extracranial types of RT administered. SRS and whole-brain RT were the most common RT to the brain, central nervous system, or leptomeningeal metastases (Table 2). The median dose of extracranial RT was 30 Gy (IQR, 30-40 Gy) in 10.0 fractions (IQR, 6.0-10.0 fractions) (Table 2).

**Table 2:** Abemaciclib and concurrent RT patterns in patients with HR+, HER2− MBC

Initial dose of abemaciclib, n (%)	
150 mg twice a day	133 (76.4)
100 mg twice a day	17 (9.8)
Other doses*	24 (13.8)
Timing of first RT initiation, n (%)	N = 174
Prior to abemaciclib initiation	23 (13.2)
At abemaciclib initiation	7 (4.0)
After abemaciclib initiation	144 (82.8)
Days from abemaciclib initiation to first overlapping RT, median (IQR)†	27.5 (5.0, 126.0)
Cumulative RT days during abemaciclib treatment duration, median (IQR)‡	14.0 (7.0, 21.0)
Duration and dose of RT	
Extracranial RT dose (Gy), median (IQR)	30 (30, 40)
RT dose fraction, median (IQR)	10.0 (6.0, 10.0)
Radiation site, n (%)	
Spine	54 (31.0)
Bone, other	33 (19.0)
Brain	30 (17.2)
Hip	24 (13.8)
Pelvis	24 (13.8)
Breast	16 (9.2)
Others	63 (36.2)
Not documented	2 (1.1)
RT for the brain, other CNS, and leptomeningeal metastases	
Stereotactic surgery	25 (14.4)
Whole-brain RT	12 (6.9)

*Abbreviations:* CNS, central nervous system; HR+, Hormone receptor-positive; HER2-, human epidermal growth factor receptor 2 negative; MBC, metastatic breast cancer; N = total number of patients; n = number of patients for the variable; RT = radiation therapy.

⁎ Include 50 mg twice a day, 150 mg every day, 200 mg twice a day, 150 mg (schedule not documented).

† Patients who were administered the course of RT on or before abemaciclib initiation were assigned a value of 0 for the number of days from index to first overlapping RT.

‡ Any RT records that overlapped each other (ie, same dates for different sites or start/end dates that overlapped then those counted as part of the same episode).

Regarding timing of first RT and initiation of abemaciclib, RT was commonly administered following abemaciclib initiation in 144 (82.8%) patients, 23 (13.2%) patients initiated RT prior to abemaciclib, and 7 (4.0%) patients started RT at the time of abemaciclib initiation. Of those initiating RT on or after abemaciclib initiation, the median time from abemaciclib initiation to first overlapping RT was 27.5 days (IQR, 5.0-126.0 days; Table 2).

### Safety outcomes

Overall, at least 1 prespecified rwAE was documented in 160 (92.0%) of patients, with majority of patients having 2 or more rwAEs, 119 (68.4%). Diarrhea (73.0%), fatigue (62.6%), and rash (27.6%) were the most common AEs (Table 3). Regarding timing of rwAEs relative to RT episode, a notable observation was that 68 (53.5%), 25 (19.7%), and 34 (26.8%) of patients reported diarrhea before, during, or after RT, respectively (Table 3). Among patients with fatigue (n = 109), 41.3% had fatigue prior to RT, 13.8% had fatigue during concurrent RT, and 45.0% had fatigue after RT. Among patients with rash (n = 48), 41.7% had rash after RT, and in patients with neutropenia (n = 40), 57.5% had neutropenia after RT (Table 3).

**Table 3:** Overall incidence and timing of rwAE in Patients with HR+, HER2− MBC who used concurrent abemaciclib and RT

rwAE*, n (%)	Overall incidence (N = 174)	rwAE prior to RT	rwAE during RT	rwAE after RT
Diarrhea	127 (73.0)	68 (53.5)	25 (19.7)	34 (26.8)
Fatigue	109 (62.6)	45 (41.3)	15 (13.8)	49 (45.0)
Rash	48 (27.6)	16 (33.3)	12 (25.0)	20 (41.7)
Neutropenia	40 (23.0)	13 (32.5)	4 (10.0)	23 (57.5)
Stomatitis	14 (8.0)	4 (28.6)	2 (14.3)	8 (57.1)
Pneumonitis	9 (5.2)	1 (11.1)	0 (0.0)	8 (88.9)
Colitis	<5 (<5)	0 (0.0)	0 (0.0)	4 (100.0)

*Abbreviations:* HR+ = hormone receptor-positive; HER2− = human epidermal growth factor receptor 2 negative; MBC = metastatic breast cancer; N = total number of patients; n = number of patients for the variable; RT = radiation therapy; rwAE = real-world adverse event.

⁎ No rwAEs reported in 14 (8.0%) patients; ≥1 rwAEs of prespecified interest reported in 160 (92.1%) patients. The majority of patients (68.4%) had 2 or more rwAEs. Therefore, the total number of events do not add up to the total patients (N = 174)

Regarding timing of RT and AE incidence, 56 and 78 patients received RT before or after diarrhea incidence, respectively (data not shown). Similarly, 62 and 53 patients received RT before or on/after experiencing fatigue, respectively (data not shown). Spine, bone (other), pelvis, hip, and brain were the most common RT sites.

### Abemaciclib dose modifications with concurrent RT

**Figure 1:**
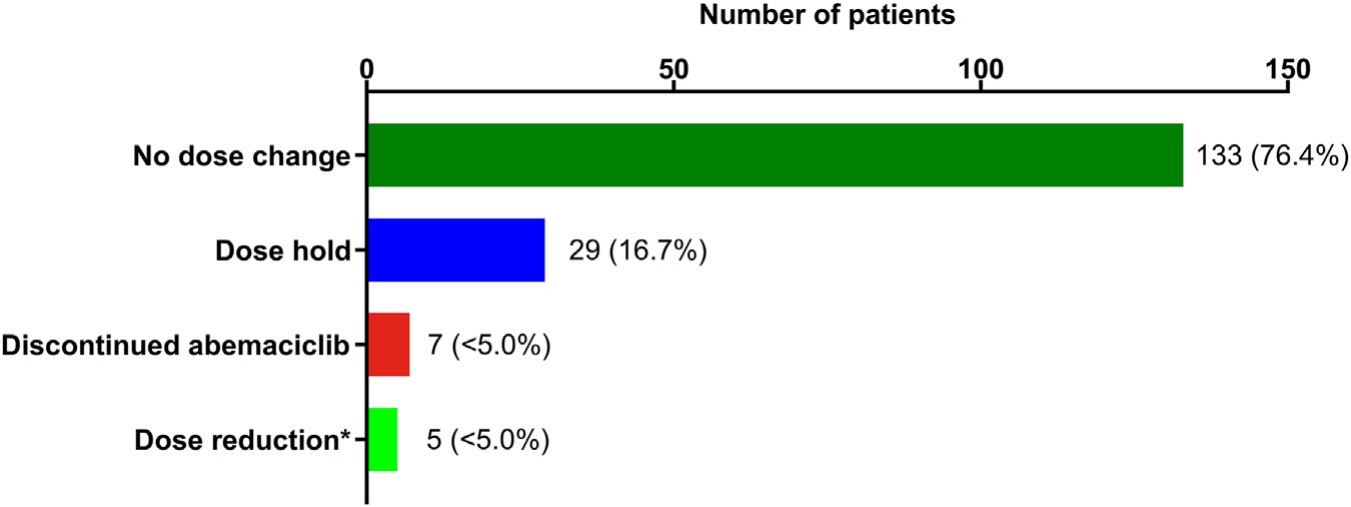
Dose modifications and interruptions in patients with hormone receptor-positive, human epidermal growth factor receptor 2 negative (HR+, HER2−) metastatic breast cancer (MBC) during concurrent abemaciclib and radiation therapy (RT). Note: *Includes one patient for whom it could not be determined if there was a dose increase or decrease.

Among patients who received concurrent abemaciclib and RT in this study, a majority of patients, 76.4% (n = 133) had no change to abemaciclib dose during concurrent RT. Twenty-nine (16.7%) patients had a dose hold during concurrent RT; of these, 15 patients had a dose hold across the entire RT episode, whereas 14 patients had a partial dose hold (some days) during the RT episode (Fig. 1).

**Figure 2:**
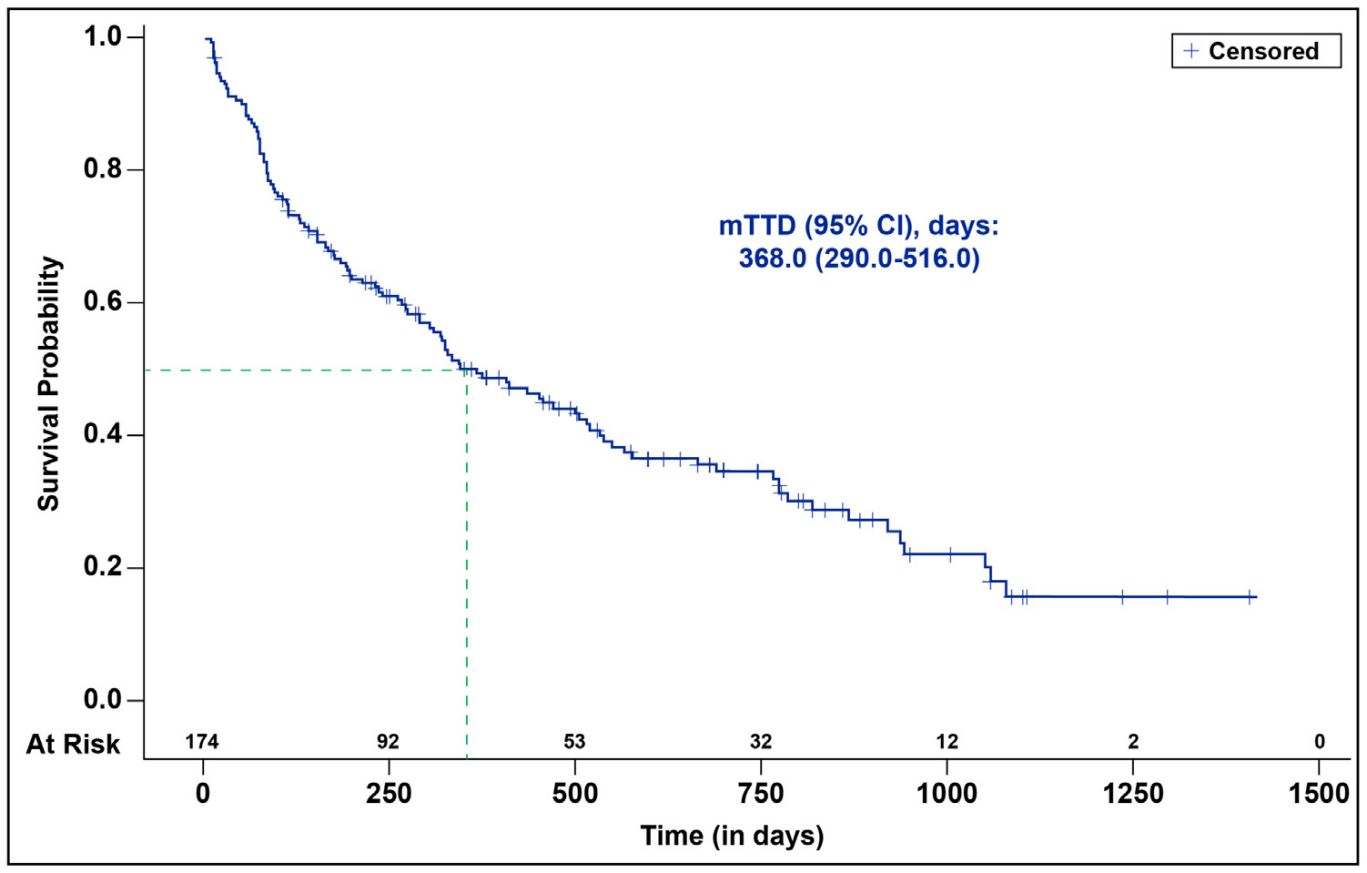
Time to abemaciclib treatment discontinuation in patients with hormone receptor-positive, human epidermal growth factor receptor 2 negative (HR+, HER2−) metastatic breast cancer (MBC) during concurrent abemaciclib and radiation therapy (RT). *Abbreviations:* CI = confidence interval; mTTD = median time to discontinuation.

At data cutoff, 61 (35.1%) patients were still on treatment and 113 (64.9%) patients discontinued abemaciclib treatment. The median TTD was 368.0 days (CI, 290.0-516.0 days; Fig. 2). Forty-nine patients (28.2%) discontinued abemaciclib due to disease progression alone; 42 (24.1%) patients discontinued due to rwAEs alone (Table 4).

**Table 4:** Reasons for abemaciclib treatment discontinuation in patients with HR+, HER2− MBC who used concurrent abemaciclib and RT (N = 174)

Reason for discontinuation of abemaciclib therapy	
Disease progression alone, n (%)	49 (28.2)
AE alone, n (%)	42 (24.1)
Disease progression + AE, n (%)	4 (2.3)
Other, n (%)*	18 (10.3)
Still on treatment, n (%)	61 (35.1)

*Abbreviations:* AE = adverse event; HR+ = hormone receptor-positive; HER2− = human epidermal growth factor receptor 2 negative; MBC = metastatic breast cancer; N = total number of patients; n = number of patients for the variable; RT = radiation therapy.

⁎ Other reasons for abemaciclib discontinuation included cancer-related symptoms not due to therapy, death, hospice, lack of data, noncancer-related medical issue, patient request, or for unknown reasons.

## Discussion

CDK4/6i as standard of care and RT are frequent treatments in the metastatic setting. However, data on concurrent use of RT and CDK4/6i are limited, and there is a lack of clinical and real-world evidence relating to safety and tolerability. This real-world study assessed the treatment patterns and safety of concurrent RT and abemaciclib in a large (n = 174) sample of US patients with HR+, HER2− MBC. The findings indicate that abemaciclib was well tolerated with over 75% of patients having no change to abemaciclib dose during the concurrent RT. Notably, <5% of patients discontinued abemaciclib during concurrent RT. The most common rwAEs were diarrhea and fatigue; over half the patients had diarrhea (54%) and over 40% had fatigue prior to RT. The median TTD of abemaciclib therapy was about a year (368 days).

Few studies have evaluated the safety of concurrent RT and abemaciclib limiting comparison of this study finding to existing literature, and the few published studies have a relatively small sample size. In Kubeczko et al,^16^ where the safety of concurrent RT and CDK4/6i was assessed (n = 46: ribociclib n = 24, palbociclib n = 17, and abemaciclib n = 5), the CDK4/6i treatment discontinuation rate was 40%.^16^ In our study, the overall abemaciclib treatment discontinuation rate due to AEs was 24.1% and abemaciclib discontinuation during concurrent RT was minimal (<5%).

During concurrent RT a significant proportion of patients (76%) did not have a dose change, 17% had a dose hold, and <5% of patients had a dose reduction during RT suggesting tolerability of abemaciclib with concurrent RT. Because approximately half of the patients with dose hold had a partial dose hold, we hypothesize that clinicians may have chosen to do this as a preventive measure. Overall, these findings align with the recent report by Ippolito et al^23^ which indicated that 10.5% (n = 2/19) of patients with metastatic or locally advanced BC who received concurrent RT with abemaciclib required a dose reduction, suggesting that concurrent RT with abemaciclib is tolerable with acceptable toxicity. The cumulative and late effects of the combination therapies on discontinuation are not in the scope of this study. However, the reasons for abemaciclib discontinuation are reported; data on discontinuation of the ET were not available (Table 4). With a median follow-up of 17.5 months, many patients discontinued abemaciclib due to disease progression or AEs. However, this study did not evaluate reasons for abemaciclib dose hold during RT, thus maybe meaning that abemaciclib discontinuation reflects a toxicity not captured by dose changes.

In this analysis, diarrhea, fatigue, rash, and neutropenia were the most commonly reported rwAEs, and this is consistent with the known safety profile of abemaciclib from clinical trials.^9^^,^^28^ The incidence of diarrhea was 73% and it was mostly reported prior to RT; fewer than one-fifth of patients had diarrhea during concurrent RT. Similarly, fatigue occurred in 62.6% of patients; <15% had fatigue during concurrent RT. The occurrence of neutropenia during RT was minimal in the present study with about 10% of patients having documented neutropenia. Other real-world studies in patients treated with abemaciclib without concurrent RT reported that diarrhea, fatigue, and neutropenia occurred in 24% to 92% of patients.^29^, ^30^, ^31^ Moreover, according to a recent Italian multicenter, observational, population-based study, in patients with HR+/HER2− advanced BC treated with abemaciclib + ET, but not RT, the discontinuation of abemaciclib due to AEs was estimated at 2.2%,^32^ whereas in an US retrospective observational study using the same database and same patient population as our study, the discontinuation rate for abemaciclib due to AE was 11.9%, but it was limited to diarrhea only.^30^

However, it was not possible to determine if any of the reported rwAEs occurred on account of abemaciclib or RT use, or both. In a retrospective analysis of patients with HR+, HER2- MBC receiving RT concurrently or within 14 days of CDK 4/6i, which had few patients receiving abemaciclib (palbociclib n = 34, abemaciclib n = 2) reported 10% (n = 3/30) of patients with grade 3 neutropenia prior to RT.^33^ In another retrospective study involving patients with MBC receiving CDK4/6i (palbociclib [n = 9], ribociclib [n = 6] and abemaciclib [n = 3]) concomitantly with palliative RT reported neutropenia in 88.8% of patients during 3 months following RT.^21^ Although the reported incidence rate of neutropenia is high, these data should be interpreted with caution due to smaller sample size (n = 18) of the study. Furthermore, each CDK4/6i exhibits distinct toxicity profiles, with palbociclib and ribociclib causing neutropenia, whereas diarrhea is the most frequent adverse event for abemaciclib.^34^ The cumulative effect of RT and the systemic treatments could not be ascertained in this study.

Previous studies assessing abemaciclib have been relatively small, making this the largest Real World study assessing tolerability of concurrent use of abemaciclib and RT (n = 174). However, there are limitations to consider in this study. Inherent to retrospective studies, selection bias, confounding and missing information need to be considered when interpreting the results. Unlike in clinical studies where grade of AEs was reported, it was not possible within this study to categorize rwAEs by grade as the use of CTCAE in clinical practice is limited. Furthermore, this study was not designed to determine the cause of rwAEs. As a result, it was not possible to determine whether the observed toxicities were due to abemaciclib or RT, or if RT exacerbated the toxicities. Only a prespecified list of rwAEs was assessed based on clinical relevance and safety profile of abemaciclib, and AEs associated with RT. Furthermore, for each patient treated with abemaciclib and overlapping RT, only the first incident of each prespecified rwAE was captured, as such, data on onset or worsening of the rwAE in patients with or without concurrent RT were not captured. Lastly, due to limited treatment information the cumulative and late effects of RT on treatment could not be ascertained.

## Conclusions

This study is the largest real-world study to assess prespecified rwAE associated with concurrent RT and abemaciclib. These findings demonstrate the tolerability of treatment with RT + abemaciclib in this cohort of HR+, HER2− MBC. Further research, ideally using prospective registries or pooled multi-institutional analyses, is needed to elucidate the relative contribution of each treatment (abemaciclib or RT) on rwAEs, including toxicity grading and RT parameters.

## Disclosures

Wambui Gathirua-Mwangi reports financial support, administrative support, article publishing charges, statistical analysis, and writing assistance were provided by Eli Lilly and Company. Sarah Rybowski reports financial support, administrative support, article publishing charges, statistical analysis, and writing assistance were provided by Eli Lilly and Company. Erich Brechtelsbauer reports financial support, administrative support, article publishing charges, statistical analysis, and writing assistance were provided by Eli Lilly and Company. Sangmi Kim reports financial support, administrative support, article publishing charges, statistical analysis, and writing assistance were provided by Eli Lilly and Company. Holly Martin reports financial support, administrative support, article publishing charges, statistical analysis, and writing assistance were provided by Eli Lilly and Company. Eileen Farrelly reports financial support and statistical analysis were provided by Eli Lilly and Company. Tasneem Lokhandwala reports financial support and statistical analysis were provided by Eli Lilly and Company. Shen Zheng reports financial support and statistical analysis were provided by Eli Lilly and Company. Wambui Gathirua-Mwangi reports a relationship with Eli Lilly and Company that includes: employment and equity or stocks. Sarah Rybowski reports a relationship with Eli Lilly and Company that includes: employment and equity or stocks. Eileen Farrelly reports a relationship with Cencora that includes: employment. Kamran Ahmed reports a relationship with Genentech that includes: funding grants. Kamran Ahmed reports a relationship with Eli Lilly and Company that includes: funding grants. Kamran Ahmed reports a relationship with Gilead Sciences Inc that includes: funding grants. Kamran Ahmed reports a relationship with Castle Biosciences Inc that includes: consulting or advisory. Erich Brechtelsbauer reports a relationship with Eli Lilly and Company that includes: employment and equity or stocks. Holly Martin reports a relationship with Eli Lilly and Company that includes: employment and equity or stocks. Sangmi Kim reports a relationship with Eli Lilly and Company that includes: employment and equity or stocks. Shen Zheng reports a relationship with TechData Service that includes: employment. Tasneem Lokhandwala reports a relationship with Cencora that includes: employment. NA If there are other authors, they declare that they have no known competing financial interests or personal relationships that could have appeared to influence the work reported in this paper.

## Appendix Supplementary materials

Supplemental Figures Image, application 1

Supplemental Tables Image, application 2

## Footnotes

Supplementary material associated with this article can be found in the online version at doi:10.1016/j.adro.2025.101992.
