# Supplementary material for: Utilization and Safety of Concurrent Use of Abemaciclib and Radiation Therapy Among Patients With HR+, HER2− Metastatic Breast Cancer in the Real-World Setting
Source: Adv Radiat Oncol. 2025 Dec 31;11(4):101992. doi: 10.1016/j.adro.2025.101992 (PMC12925158; doi:10.1016/j.adro.2025.101992)
Supplement: Supplemental Figures [file mmc1.docx]

**
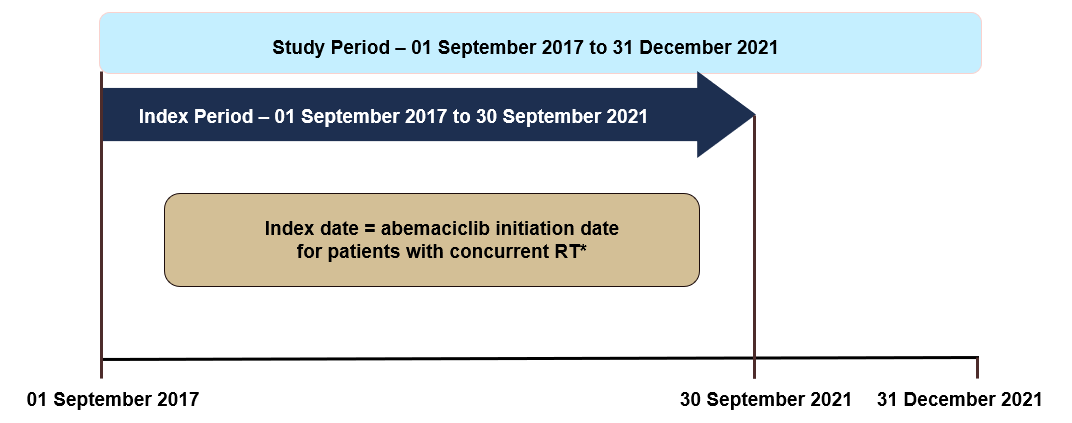
Figure E1. Study Design**

**Abbreviations**: RT, radiation therapy; rwAE, real-world adverse event

**Note: ***Concurrent RT was defined as receipt of any RT with at least 1 day of overlap with the abemaciclib treatment span.

rwAEs were captured during abemaciclib treatment span up to and including 30 days after abemaciclib treatment discontinuation.

**Figure E2: Patient attrition**

N=691,435

Had evidence of stage IV or recurrent MBC with a metastatic diagnosis date on or after January 01, 2011 and had evidence of HR+, HER2- MBC^2,3,4^

Patients with evidence of breast cancer diagnosis^1^ and ≥ 2 documented clinical encounters on different days in flatiron network on or after January 01, 2011

Age ≥18 years at start of first abemaciclib contain

ning LOT in mBC settings and initiated treatment with abemaciclib as monotherapy or combination therapy on or after January 09, 2017

n=1,500

n=2,350

Evidence of receipt of radiotherapy concurrently with abemaciclib in the metastatic setting

No evidence of HR+, HER2- status on the closest valid test any time prior to or within 30 days following the index date; diagnosis of primary malignancies other than MBC while being treated for MBC; Patients receiving HER2+ targeted therapy as part of the index LOT; Treated with <7 day supply of abemaciclib at treatment initiation^5^; Patients with <90 days of follow-up from index date based on their last follow-up date in the study timeframe

(n=1242)

Patients receiving abemaciclib concurrent with RT for MBC or within 14 days pre-/post- the abemaciclib span

n=242

Patients with erroneous data (n=13); Evidence of receipt of radiotherapy within 14 days pre-/post- the abemaciclib span but no overlap with abemaciclib (n=55)

Overall population n=174

^1^ICD-9-CM 174.x or 175.x; ICD-10-CM: C50x; ^2^HR+ was defined as any ER or PR positive test before or up to 60 days after the metastatic diagnosis date; ^3^HER2- was defined by any HER2- test (IHC negative (0-1+), FISH negative /not amplified, Negative NOS, NGS, Negative (ERBB2 not amplified) and the absence of positive test (IHC positive (3+), FISH positive/amplified, positive NOS, NGS positive (ERBB2 amplified) before or up to 60 days after the metastatic diagnosis date; ^4^Biomarker test date was defined using the lattr of the biomarker result date and specimen collected date. Tests with null test dates were included; ^5^Days during which treatment was held were included in the days supply if the hold was ≤60 days.

**Abbreviations:** ER, estrogen receptor; FISH, fluorescence in situ hybridization; HR+, HER2-, Hormone receptor positive, human epidermal growth factor receptor 2 negative; ICD-9-CM, International Classification of Diseases, Ninth Revision, Clinical Modification; ICD-10-CM, International Classification of Diseases, Tenth Revision, Clinical Modification; IHC, immunohistochemistry; LOT, line of treatment; MBC, metastatic breast cancer; N, total number of patients; n, number of patients in each step; NGS, next generation sequencing; NOS, not otherwise specified; PR, progesterone receptor; RT, radiation therapy
