# Supplementary material for: Utilization and Safety of Concurrent Use of Abemaciclib and Radiation Therapy Among Patients With HR+, HER2− Metastatic Breast Cancer in the Real-World Setting
Source: Adv Radiat Oncol. 2025 Dec 31;11(4):101992. doi: 10.1016/j.adro.2025.101992 (PMC12925158; doi:10.1016/j.adro.2025.101992)
Supplement: Supplemental Tables [file mmc2.docx]

**Table E1. Baseline Characteristics of Patients by abemaciclib treatment regimens (N = 174)**

| Variables | Abemaciclib + AI  n = 59 (33.9%) | | | | Abemaciclib + Fulvestrant  n = 83 (47.7%) | | Abemaciclib Monotherapy  n = 21 (12.1%) |
| --- | --- | --- | --- | --- | --- | --- | --- |
| Age (in years), Median (IQR) | 58.0 (50.0, 67.0) | | | | 64.0 (56.0, 73.0) | | 66.0 (56.0, 72.0) |
| Sex, n (%) |  | | | |  | |  |
| Female | 59 (100.0) | | | | 83 (100.0) | | 21 (100.0) |
| Race, n (%) |  | | | |  | |  |
| White | 31 (52.5) | | | | 53 (63.9) | | 13 (61.9) |
| Black | 8 (13.6) | | | | 11 (13.3) | | <5 (≥5.0) |
| Other^a^ | 15 (25.4) | | | | 13 (15.6) | | <5 (≥5.0) |
| Missing/unknown | ≤5 (≥5.0) | | | | 6 (7.2) | | <5 (≥5.0) |
| Ethnicity, n (%) |  | | | |  | |  |
| Hispanic | 8 (13.6) | | | | 7 (8.4) | | <5 (≥5.0) |
| US geographical region, n (%) |  | | | |  | |  |
| Midwest | <5 (<5.0) | | | | 13 (15.7) | | <5 (≥5.0) |
| Northeast | | 12 (20.3) | | 15 (18.1) | | <5 (≥5.0) | |
| South | | 32 (54.2) | | 34 (41.0) | | 9 (42.9) | |
| West | | 9 (15.3) | | 17 (20.5) | | ≤5 (≥5.0) | |
| Unknown | | <5 (≥5.0) | | <5 (<5.0) | | <5 (≥5.0) | |
| Practice type, n (%) | |  | |  | |  | |
| Academic | | <5 (≥5.0) | | <5 (<5.0) | | 0 (0.0) | |
| Community | | 54 (91.5) | | 81 (97.6) | | 15 (71.4) | |
| Unknown | | <5 (<5.0) | 0 (0.0) | | | 6 (28.6) | |
| Menopausal status, n (%) | |  |  | | |  | |
| Pre/peri-menopausal | | 12 (20.3) | ≤5 (≥5.0) | | | 0 (0.0) | |
| Post-menopausal | | 43 (72.9) | 74 (89.2) | | | 19 (90.5) | |
| Unknown | | <5 (≥5.0) | <5 (<5.0) | | | <5 (≥5.0) | |
| Follow-up time (in months), Median (IQR) | | 17.3 (7.3, 26.5) | 18.3 (11.3, 26.7) | | | 18.1 (15.1, 27.8) | |

**Abbreviations:** AI, aromatase inhibitors, IQR, interquartile range; N, total number of patients; n, number of patients in the subgroup.

Note: Eleven (6.3%) of patients used abemaciclib plus other therapies such as included anastrozole, cyclophosphamide, doxorubicin, letrozole, exemestane, palbociclib, and tamoxifen

^a^Includes Asians, American Indian/Alaska Native, Hawaiian or Pacific Islander.

^b^ Female >60 years at index were assigned as postmenopausal and if <60 years, if they underwent bilateral oophorectomy before or up to 30 days of using any abemaciclib-containing therapy.

**Table E2: Radiation site categories of patients by abemaciclib treatment regimens**

| Radiation site categories, n (%) | All Abemaciclib  N=174 | Abemaciclib + AI  n = 59 | Abemaciclib + Fulvestrant  n = 83 | Abemaciclib Monotherapy  n = 21 |
| --- | --- | --- | --- | --- |
| All bone | 112 (64.4) | 35 (59.3) | 51 (61.5) | 18 (85.7) |
| Brain and other CNS (Brain, CNS, leptomeningeal) | 40 (23.0) | 12 (20.3) | 22 (26.5) | 3 (14.3) |
| Visceral (liver, lung) | 10 (5.8) | 7 (11.9) | 3 (3.6) | 0 (0) |
| Other (Breast, lymph nodes, other) | 32 (18.4) | 12 (20.3) | 13 (15.7) | 4 (19.1) |
| Not documented | 2 (1.2%) | 0 (0) | 2 (2.4) | 0 (0) |

**Abbreviations:** AI, aromatase inhibitors, CNS, central nervous system; N, total number of patients; n, number of patients in the subgroup.
